# Three-stage laparoscopic surgery in a morbidly obese patient with Hinchey III diverticulitis: a case report

**DOI:** 10.1186/s40792-019-0588-7

**Published:** 2019-02-15

**Authors:** Takako Tanaka, Yoshiaki Kita, Shinichiro Mori, Kenji Baba, Kan Tanabe, Masumi Wada, Yusuke Tsuruda, Kiyonori Tanoue, Shigehiro Yanagita, Kosei Maemura, Shoji Natsugoe

**Affiliations:** Department of Digestive Surgery, Breast and Thyroid Surgery, Kagoshima University Graduate School of Medical and Dental Sciences, Sakuragaoka 8-35-1, Kagoshima, 890-8520 Japan

**Keywords:** Perforated diverticulitis, Hinchey III diverticulitis, Three-stage surgery, Obesity, Laparoscopic surgery

## Abstract

**Background:**

Perforated diverticulitis with purulent peritonitis (Hinchey III diverticulitis) has traditionally been treated with a Hartmann’s procedure in order to avoid the considerable postoperative morbidity and mortality associated with one-stage resection and primary anastomosis. Although there have been reports regarding laparoscopic lavage as the initial treatment of perforated Hinchey III diverticulitis, a formal treatment strategy has not been established yet. We performed a three-stage surgery, including laparoscopic lavage and drainage with diverting ileostomy (first stage), laparoscopic sigmoidectomy (second stage), and ileostomy closure (third stage) in a morbidly obese patient with Hinchey III diverticulitis.

**Case presentation:**

A 31-year-old man who presented with abdominal pain was diagnosed with perforated diverticulitis and sent to our hospital for evaluation. He had morbid obesity (body mass index (BMI) 50 kg/m^2^), acute renal failure, and uncontrolled diabetes. We performed an emergency operation including laparoscopic lavage and drainage with a diverting ileostomy for this case of Hinchey III diverticulitis. Fifteen months after the first-stage surgery, we performed laparoscopic sigmoidectomy as the second stage. Finally, 5 months later, we performed ileostomy closure. The patient recovered without significant complications.

**Conclusion:**

Three-stage surgery including early laparoscopic lavage and proximal diversion for morbidly obese, comorbid patients with Hinchey III diverticulitis may be indicated in the acute phase to avoid perioperative complications and permanent colostomy creation.

## Background

**Fig. 1:**
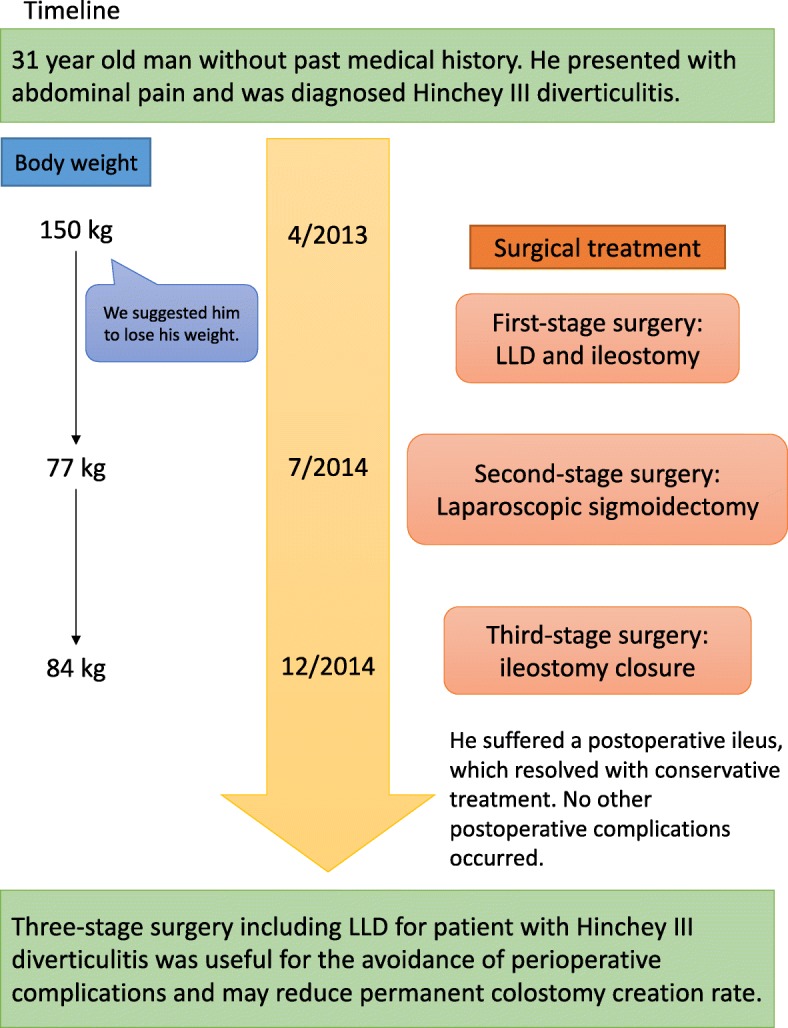
Timeline of this case report

Diverticulitis of the colon occurs quite frequently in developed countries, and an increasing prevalence of the disease has been reported in Japan [1]. That is often classified according to the Hinchey classification scheme [2]. Hinchey III and IV diverticulitis frequently require early operative intervention to avoid progression to advanced peritonitis and even mortality. The traditional surgical treatment for this group of patients has been the Hartmann’s procedure [3]. However, considerable morbidity has been reported after this procedure [4]. Beginning in the 1990s, laparoscopic lavage and drainage (LLD) was proposed for patients with Hinchey III diverticulitis [5], with the benefit of being a minimally invasive modality. However, most patients with Hinchey III diverticulitis will not be definitively treated with LLD alone, and will occasionally require reintervention [6]. Moreover, according to a recent phase III trial, the most effective treatment strategy is not clear [3, 7–9]. Herein, we present a case of three-stage surgery in a morbidly obese patient with Hinchey III diverticulitis (Fig. 1). In this case, staged LLD with diverting ileostomy, followed by laparoscopic sigmoidectomy, and ultimately ileostomy closure was performed to minimize perioperative risk and complications and to avoid the possibility of a permanent colostomy. Moreover, we discuss tips and considerations with reference to the surgical literature in regards to optimal treatment strategy for Hinchey III diverticulitis.

## Case presentation

**Fig. 2:**
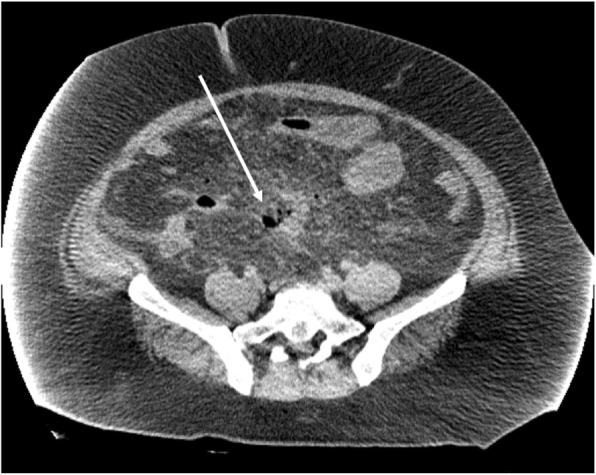
Abdominal computed tomography showing free air around the sigmoid colon

A 31-year-old Asian man who was a schoolmaster presented with lower abdominal pain and was diagnosed with an acute perforation of the sigmoid colon by computed tomography (CT) at an outside hospital (Fig. 2). He has neither past medical history nor family history. He was morbidly obese, weighing 150 kg (BMI 50 kg/m^2^), and laboratory data showed acute renal failure (creatinine 2.59 mg/dL, blood urea nitrogen 26.8 mg/dL) and uncontrolled diabetes (DM) (blood glucose level 345 mg/dL). After initial outside admission into the intensive care unit (ICU), he was transferred to our hospital and consented to undergo emergency surgery.

**Fig. 3:**
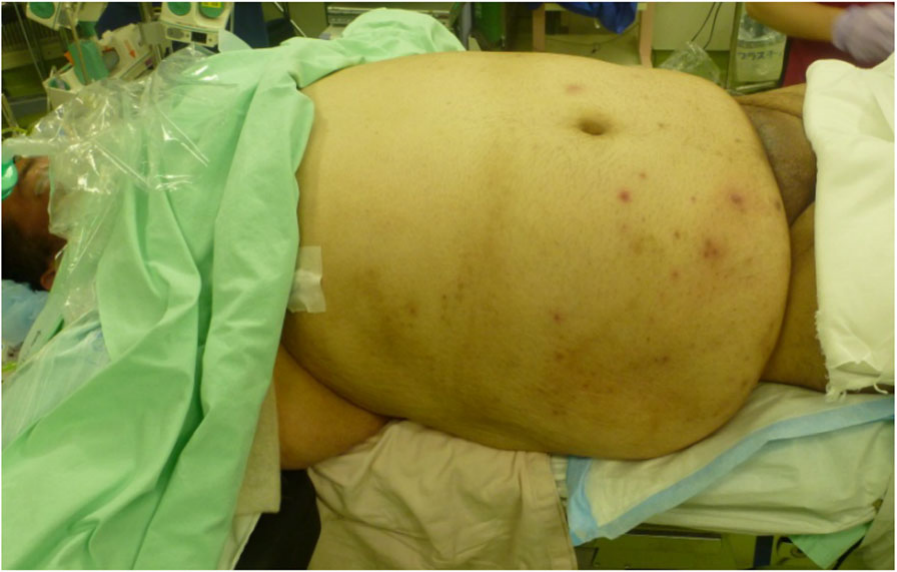
First-stage surgery. The patient was morbidly obese and difficult to rotate on the operating table

**Fig. 4:**
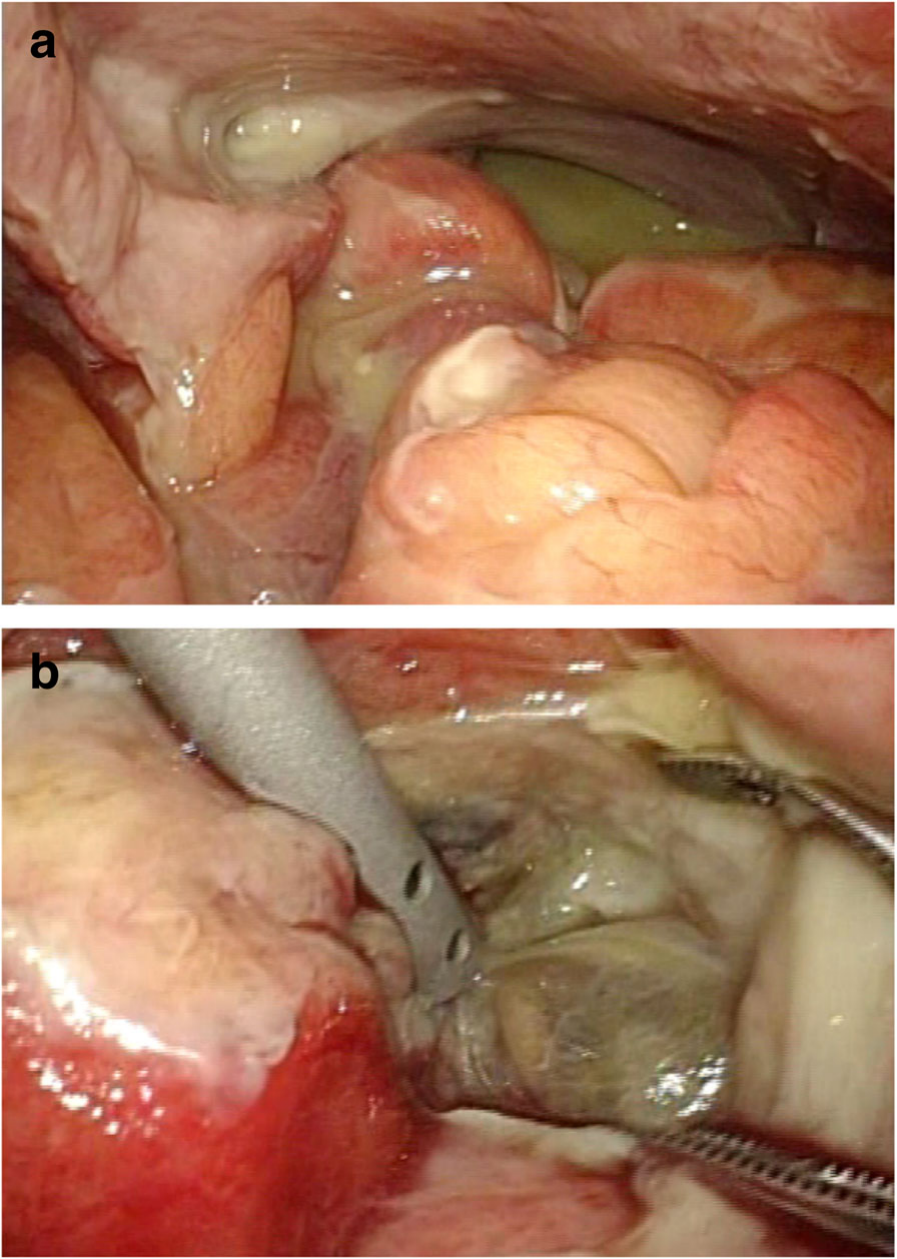
Initial operative findings. **a** Purulence in the rectovesical pouch. **b** Abscess cavity of the mesentery of the sigmoid colon

The patient was placed in the supine position and was induced under general anesthesia (Fig. 3). A 12-mm trocar for a 10-mm flexible laparoscope was inserted through the umbilicus using an open technique. Pneumoperitoneum was maintained at 12 mmHg with carbon dioxide. Next, one 12-mm and three 5-mm long trocars were placed under laparoscopic visualization, and the abdominal cavity was explored. We performed LLD and diverting ileostomy as the first-stage surgery. After adhesions of the peritoneum and greater omentum were dissected from the pelvis, purulence was drained from the rectovesical pouch (Fig. 4a). A large amount of purulence was also drained from the mesentery after exposure of an abscess cavity (Fig. 4b). After peritoneal lavage using 36 L of saline, no gross fecal contamination was noted. After placement of drainage tubes into the abscess cavity, the right and left subphrenic spaces, the right pararectal fossa, and the rectovesical pouch, we created a diverting loop ileostomy. The operation time was 372 min and blood loss was 240 mL without any major complications during the first operation. He started oral intake from post-operation day (POD) 3. He was transferred to another hospital to receive medical treatment with drainage tube and wound in POD17. The drainage tube was removed in POD33. There were no complications after surgery in all hospitalizations.

**Fig. 5:**
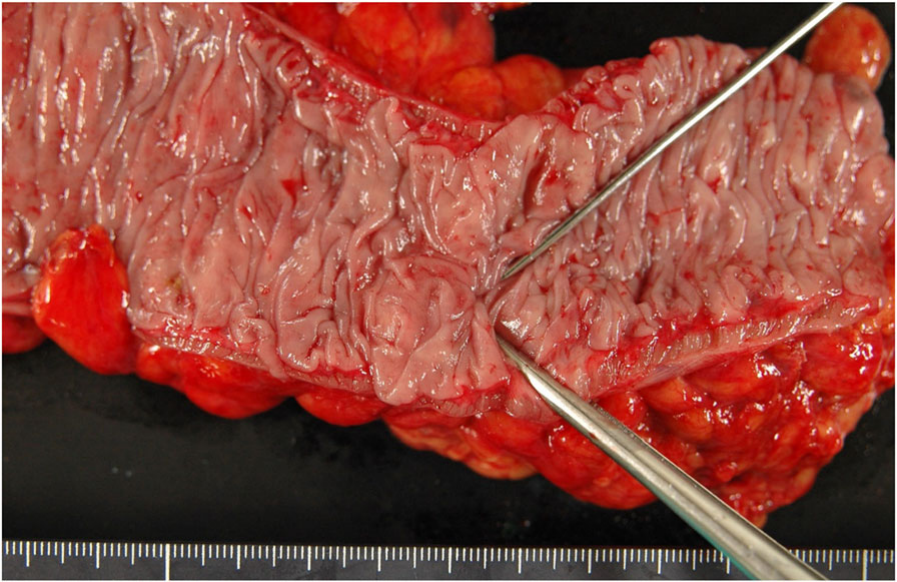
Macrograph of the resected sigmoid colon showing healed diverticulitis and a large diverticulum

One year later, the patient was seen in follow-up after losing approximately 70 kg. He safely and successfully lost his weight by the educational admission to the diabetic tract medicine. Barium enema examination revealed numerous diverticulum of the sigmoid colon. We performed laparoscopic sigmoidectomy in the lithotomy position as the second-stage surgery. After inserting six trocars, the abdominal cavity was explored. The sigmoid colon was densely adherent to the pelvic cavity, and an incisional hernia around the ileostomy was detected without surrounding adhesions. After displacing the small intestine towards the right upper quadrant of the abdomen, a medial to lateral approach for the mesenteric dissection was undertaken. The specimen was extracted from the abdomen through the umbilical incision. An intra-corporeal double stapling technique was used to complete the anastomosis. At the end of the operation, a drain was inserted behind the colonic anastomosis. Pathological examination revealed diverticula with panniculitis of the sigmoid colon (Fig. 5). He was discharged 7 days postoperatively after an uneventful hospital course.

Five months after the second-stage surgery, we performed ileostomy closure and incisional hernia repair as the third-stage surgery. He suffered a postoperative ileus, which resolved with conservative treatment. No other postoperative complications occurred.

## Discussion

Although laparoscopic lavage and drainage has gained wider acceptance as an alternative to primary resection for Hinchey III diverticulitis, its use for frank diverticular perforation is still controversial, and further evidence is needed to guide treatment strategies [10]. Recently, McDermott et al. reported that LLD and antibiotics as an initial therapeutic strategy potentially avoid radical colon resection and the possibility of a permanent colostomy for Hinchey III diverticulitis [11]. However, considering the high frequency of recurrence of symptoms within 2–6 months after LLD [6], as well as the occult neoplasia which can occur in 10% of patients presenting with a sigmoid perforation [12], LLD should be viewed only as a damage control procedure; eventual colectomy after LLD is indicated for Hinchey III diverticulitis.

In order to compare LLD to open colonic resection as the first procedure of choice, several randomized controlled trials have been completed. In the DILALA trial, LLD was compared to colon resection and stoma creation for Hinchey III diverticulitis in Sweden and Denmark. After 39 patients undergoing laparoscopic lavage and 36 patients undergoing the Hartmann’s procedure were compared, morbidity and mortality did not differ between the groups. It was concluded that LLD for Hinchey III diverticulitis was feasible and safe [3, 7]. In the Ladies trial in Belgium, Italy, and the Netherlands, which was similar in design to the DILALA trial, the authors concluded that LLD is not superior to radical sigmoid colectomy except when compared to the Hartmann’s procedure [8]. Major morbidity arose in 30 of 45 LLD patients (67%) and in 25 of 42 sigmoid colectomy patients (60%); this was not a statistically significant difference. There was no obvious difference in 12-month mortality, with four patients dying after lavage and six patients dying after sigmoidectomy (*p* = 0.43).

The SCANDIV randomized clinical trial in Sweden and Norway compared LLD and colon resection for Hinchey III and IV diverticulitis [9]. This study set major postoperative complications within 90 days as the primary endpoint. The primary endpoint occurred in 31 of 101 patients (30.7%) in the laparoscopic lavage group and in 25 of 96 patients (26.0%) in the colon resection group, with no significant difference. Additionally, there was no significant difference in mortality at 90 days, reoperation rate, length of postoperative hospital stay, and quality of life between the groups. Although inclusion of patients with Hinchey IV diverticulitis is controversial, the findings of this RCT suggest a role for LLD in patients with advanced diverticulitis.

In the present case, with a morbidly obese patient who presented with acute renal failure and uncontrolled diabetes, we felt that LLD and diverting ileostomy as initial treatment was the most appropriate strategy, both to minimize the invasiveness of the initial surgery and to avoid permanent colostomy, as well as to reduce the incidence of perioperative complications. It allowed the patient to stabilize and receive early and aggressive postoperative management in the ICU. While the appropriateness of a diverting ileostomy in this setting is still unknown due to the small cohorts in the randomized trials outlined above, LLD with diverting ileostomy usually leads to faster postoperative initiation of oral intake and minimizes postsurgical adhesions for the second-stage colonic resection. However, this multi-staged intervention is associated with increased cost and use of resources. In the present case, staging the surgeries was beneficial as it allowed the patient to lose significant weight in between procedures with enough time to increase compliances, potentially reducing postoperative complications. Furthermore, while we did not perform ileostomy closure during the second-stage surgery, addition of a third stage did not add significantly to the burden on the patient. The proper timing of ileostomy closure varies depending on the clinical scenario. Selection of a three-staged approach for Hinchey III diverticulitis requires careful and appropriate patient selection prior to widespread adoption. Basically, we performed a three-staged approach for all patients with Hinchey III diverticulitis. We anticipate the clinical trial from a pilot study to accumulate evidence and confidence with this three-stage approach. While a three-staged surgical strategy seemed to be an acceptable modality in the present case, its safety and feasibility for patients with Hinchey III diverticulitis should be assessed in randomized trials.

## Conclusion

Three-stage surgery including an early laparoscopic lavage component for morbidly obese patients with Hinchey III diverticulitis may be useful for the avoidance of perioperative complications and may reduce permanent colostomy creation rate. This novel strategy is a safe alternative surgical approach in patients with Hinchey III diverticulitis.

## Abbreviations

BMI: Body mass index
CT: Computed tomography
DM: Diabetes mellitus
ICU: Intensive care unit
LLD: Laparoscopic lavage and drainage
POD: Post-operation day
